# A new permanent cell line derived from the bank vole (*Myodes glareolus*) as cell culture model for zoonotic viruses

**DOI:** 10.1186/1743-422X-8-339

**Published:** 2011-07-05

**Authors:** Sandra S Essbauer, Ellen Krautkrämer, Sibylle Herzog, Martin Pfeffer

**Affiliations:** 1 Bundeswehr Institute of Microbiology, Neuherbergstr. 11, D-80937 Munich, Germany; 2 Nephrology, University of Heidelberg, Im Neuenheimer Feld 162, 69120 Heidelberg, Germany; 3 Institute of Virology, Department Veterinary Medicine, Justus-Liebig-University, Frankfurter Str. 107, 35392 Giessen, Germany; 4 Institute of Animal Hygiene and Veterinary Public Health, Centre of Veterinary Public Health, University Leipzig, An den Tierkliniken 1, 04103 Leipzig, Germany

## Abstract

**Background:**

Approximately 60% of emerging viruses are of zoonotic origin, with three-fourths derived from wild animals. Many of these zoonotic diseases are transmitted by rodents with important information about their reservoir dynamics and pathogenesis missing. One main reason for the gap in our knowledge is the lack of adequate cell culture systems as models for the investigation of rodent-borne (robo) viruses *in vitro*. Therefore we established and characterized a new cell line, BVK168, using the kidney of a bank vole, *Myodes glareolus, *the most abundant member of the *Arvicolinae *trapped in Germany.

**Results:**

BVK168 proved to be of epithelial morphology expressing tight junctions as well as adherence junction proteins. The BVK168 cells were analyzed for their infectability by several arbo- and robo-viruses: Vesicular stomatitis virus, vaccinia virus, cowpox virus, Sindbis virus, Pixuna virus, Usutu virus, Inkoo virus, Puumalavirus, and Borna disease virus (BDV). The cell line was susceptible for all tested viruses, and most interestingly also for the difficult to propagate BDV.

**Conclusion:**

In conclusion, the newly established cell line from wildlife rodents seems to be an excellent tool for the isolation and characterization of new rodent-associated viruses and may be used as *in vitro-*model to study properties and pathogenesis of these agents.

## Background

About 800 out of the ~1.400 known human pathogens are of zoonotic origin [1,2]. Recent analyses of emerging infections revealed that three-fourth of the zoonotic agents originate from wild animal reservoirs [2]. Rodents and other small mammals are associated with zoonotic infectious agents belonging to different taxa, i.e. RNA viruses, DNA viruses, bacteria and parasites that induce sometimes severe diseases in humans. Many of these pathogens are associated with a particular rodent reservoir host, thus showing diverse geographical and biotope usage, and often adapted transmission cycles. The occurrence of human infections by rodent-borne pathogens is therefore influenced by the geographical distribution, abundance and the prevalence in the reservoirs [3]. Rodents are known to be the reservoir hosts for a variety of zoonotic viruses which are sometimes tedious to isolate or cultivate in usually applied cell lines. Bank voles (*Myodes glareolus*) are geographically distributed in central Europe from France to Scandinavia to Lake Baikal, in the South to Northern Spain and Italy, the Balkans, Western and Northern Turkey, as well as in Britain and south-west Ireland [4]. So far bank voles have been shown to be an important reservoir or/and host for many rodent-borne agents [3]. These voles are the reservoir for Puumala virus (genus Hantavirus, family *Bunyaviridae*), the main hantavirus species in Europe with approximately 3400 human clinically apparent infections recorded in Germany alone since 2001 (for review see [3,5]). Recent serological investigations showed that bank voles in South Germany are the main rodent hosts for cowpox virus (CPXV, genus Orthopoxvirus, family *Poxviridae*) that induces pocks and exanthema in a broad host spectrum including humans [6]. However, despite of the increasing reports of evidence of CPXV in rodents in several European countries there exists to the authors' knowledge no published virus isolate from wildlife rodents (for review see [7]). In the last years several bank vole-associated agents have been newly discovered. These include e.g. Ljunganvirus (genus *Parechovirus*, family *Picornaviridae*) in bank voles in Sweden that is not easy to reliably grow in cell-culture [8,9] and identification of novel herpes viruses (*Myodes glareolus *cytomegalovirus 1 and *Myodes glareolus *rhadinovirus 1, family *Herpesviridae*) from bank voles in Germany that could not be isolated yet [10].

Further, for some viruses small mammals are discussed as reservoirs, however they cannot easily be propagated in cell lines *in vitro*. One of these candidates is Borna disease virus (BDV, genus *Bornavirus*, family *Bornaviridae*) [11]. The presence of this virus in tissues of shrews was described in Switzerland [12], but further data are still lacking. BDV induces a severe T-cell-mediated meningoencephalitis in animals like horse, sheep and rabbit, and the pathogenicity for humans is controversially discussed among scientists. However, trials to isolate BDV from naturally affected animals on different cell lines with different species origin were not successful. So far, only rabbit embryonic brain cells (REB) are well susceptible for isolation of BDV and for infectivity assays. But still, here the virus propagation is restricted by the limited subcultivation cycles of REB cells for only 7-10 cell passages [13].

Preparation of primary bank vole cells for the propagation of PUUV has already been described [14] but - to the authors' knowledge - a permanent cell line of *M. glareolus *has not been established. Therefore, we herein describe the establishment and characterization of a permanent cell line derived from bank vole (*Myodes glareolus*) kidneys. Representatively, we have chosen nine lab-adapted viruses out of six different virus families that are either rodent-borne (Cowpox, Vaccinia, Puumala virus) or suspected to be rodent-associated (Borna disease virus, Vesicular stomatitis virus, Sindbis virus, Inkoo virus) or at least pathogenic for lab mice and without known animal reservoir (Pixuna virus, Usutu virus,). A summary of these viruses is given in table 1. We could show that BVK 168 allows the propagation of known rodent-borne and rodent-associated viruses but leads to a non-productive replication of a lab-adapted Puumalavirus strain.

**Table 1:** Viruses used for the infectability analysis of the established bank vole cell line

Family	Genus	Group, virus	Strain	Original cell line	Pathogenic for humans	Role of rodents	Pathogenic for other vertebrates
*Rhabdoviridae*	*Vesiculovirus*	Vesicular stomatitis virus	India	Vero B4	+	neurotrop; deer mouse may transmit VSV ^1^	+, cattle, pigs
*Poxviridae*	*Orthopoxvirus*	Vaccinia virus	Munich 1	Ma104	+	host ^2^	+, cattle
*Poxviridae*	*Orthopoxvirus*	Cowpox virus	81/01	Ma104	+	host ^3^	+, broad host range
*Togaviridae*	*Alphavirus*	Sindbis virus	Australia C377	Vero B4	+	neuropathogenic in suckling lab mice ^4^	birds
*Togaviridae*	*Alphavirus*	Pixuna virus	BeAr 35645	Vero B4	+	neuropathogenic for lab rats and mice ^5^	+, horses
*Bunyaviridae*	*Orthobunyavirus*	Inkoovirus	TN-98-5085*	Vero B4	+	pathogenic for lab mice ^6^	unknown°
*Bunyaviridae*	*Hantavirus*	Puumalavirus	Vranica	Vero E6	*+*	*M. glareolus *serve as PUUV reservoir ^7^	-
*Flaviviridae*	*Flavivirus*	Usutu virus	0679/2006	Vero B4	+	neuropathogenic for suckling mice ^8^	+, birds
*Bornaviridae*	*Bornavirus*	Borna disease virus	BDV H24 adapted to Lewis rats	rabbit embryonic brain cells	(+)	neuropathogenic for Lewis rats ^9 ^and white mice ^10^	+, horses, sheep, rabbits

* Pfeffer unpublished data; ^° ^high seroprevalences found in large animals

References: ^1 ^[15]; ^2 ^[16,17]; ^3 ^for review see [7]; ^4 ^[18]; ^5 ^[19]; ^6 ^[20,21]; ^7 ^[22]; ^8 ^[23]; ^9 ^[24]; ^10 ^[25]

## Material and Methods

### Origin of animals

Bank voles were trapped in January 2002 with Sherman traps in a garden in Grafrath, administrative district Fürstenfeldbruck, South Germany. Voles were anaesthetized by CO_2 _and killed through exsanguinations. All procedures including the use and treatment of animals were in agreement with the German Law of Animal Protection. Tissues were collected for screening for zoonotic agents and the preparation of cell lines. Romanowsky-Giemsa staining was performed as described in detail by [26].

### Preparation of bank vole cell line

One kidney (0.9 g) of the adult male bank vole No. 168 was taken for the preparation of cells and directly transferred into 2 ml phosphate-buffered saline (PBS) containing 10x antibiotic-antimycotic solution (AAS, Invitrogen, Karlsruhe, Germany). The organ was roughly minced using sterile siccors and digestion of tissues was carried out by adding 1 ml 0.05% trypsin for 35 min. The trypsinate was further homogenized by repeated aspiration using a 5 ml-syringe. Cell debris was removed by centrifugation at 400 g for 5 minutes. After re-suspension of the cells in 3 ml MEM containing 10% fetal calf serum (FCS) and 10x AAS, these were inoculated in a 25 cm^2 ^flask. For the first 5 weeks partial (50%) medium change was performed twice a week. From week six on, every 4 up to 7 days subcultures were gained by trypsination of out-grown cells with a split ratio of 1:3 using standard protocols [27]. Established cell cultures were designated "BVK168" (bank vole kidney No. 168).

### Confirmation of cell line origin from bank voles

Nucleic acids (NA) were extracted from cell lines (22^th ^passage) using the RNeasy kit (Qiagen, Hilden, Germany) according the manufacturer's instructions. Species determination of the bank vole line was confirmed using a PCR specific for the mitochondrial cytochrome B (cyt B) gene (for details see [28]) followed by direct sequencing of the purified PCR products. The GenBank accession number for the derived partial cyt B gene is FJ528598.

### Cell contamination assays

The bank vole No. 168 was serologically investigated for hantavirus and flavivirus antibodies using Biochips (Euroimmun, Lübeck, Germany) and for orthopox virus (OPV) antibodies using a serum neutralization assay [6]. NA isolated from tissues of the vole as described above was further screened by a PCR specific for borrelia (ears) and real-time PCR specific for leptospira (second kidney) using protocols described elsewhere [29-31]. A mycoplasma assay was performed at regular intervals - every 4^th ^subculture from the 6^th ^subculture on - using the Venor^®^GeM as described by the manufacturer (Minerva Biolabs, Berlin, Germany). Detection of contaminations was further analysed sending samples of the 22^th ^passage of BVK168 cell lines to the Multiplex cell Contamination Test (McCT) Service, Heidelberg, Germany (http://www.multiplexion.de). The test there includes a multiplex PCR assays for Squirrel Monkey Retrovirus (SMRV), Mycoplasma, Human Papillomavirus 18 (HeLa), Adenoviruses 1, 2, 5, 6, Hepatitis B virus, Human Herpes virus 1-8, SV40 (VP1, VP3, Tag sequences). Further, this assay provides detection of cross-contamination of cell lines with human, African Green Monkey-, house mouse (*Mus musculus*), rat, Chinese hamster, canine, feline, rabbit and Guinea pig cells, and detection of human/primate Y-chromosomes.

### Investigation of cell surface markers by immunofluorescence

In order to investigate whether BVK168 cell line is of the epitheloid or fibroblastoid type, different type-specific cell surface markers were examined. For immunofluorescence, BVK168 cells grown on coverslips were fixed with acetone or with 3% paraformaldehyde-PBS and incubated with the following primary antibodies and appropriate Cy3-conjugated secondary antibodies: mouse monoclonal vimentin clone VIM3B4 (Zytomed/Invitrogen, Karlsruhe), mouse E-cadherin clone 4A2C7 (Zytomed/Invitrogen, Karlsruhe), mouse cytokeratin 18 clone RGE53 (Millipore, Schwalbach), mouse β-catenin clone E-5 (Santa Cruz, Heidelberg), rabbit Zonula Occludens ZO-1 clone Z-R1 (Zytomed/Invitrogen, Karlsruhe), rabbit occludin (Zytomed/Invitrogen, Karlsruhe), goat von Willebrand factor vWF F15 (Santa Cruz, Heidelberg), rabbit S100A (Dako Cytomation, Hamburg), mouse CD31 JC70A (Dako Cytomation, Hamburg). Images were then taken with a Nikon DS-Qi1Mc quantitative black-and-white charge-coupled device camera attached to a Nikon Eclipse 80i upright microscope (Nikon, Düsseldorf, Germany).

### Virus infection of BVK168 cells

In order to see whether BVK168 cell line could be useful in attempts to isolate or propagate viruses, we selected nine vector-transmitted viruses with known or suspected rodent reservoir, or known rodent-borne viruses belonging to six virus families (see table 1) for the analysis of the susceptibility of the new established cell line. Usutuvirus was kindly provided by N. Nowotny.

#### Rhabdo-, Orthopox-, Alpha-, Orthobunya- Flaviviruses

BVK168 cell lines were infected with 0.5 ml of the respective viruses by adsorbing for 1 hour at 37°C. Cells were inoculated in MEM containing 2% fetal calf serum (FCS) and checked daily for cytopathogenic effects (CPE). Infected BVK168 cells were frozen after prominent CPE was obtained or ultimately 7 days post infectionem (p.i., 1^st ^passage). After freeze-thawing, a 0.5 ml-aliquot of the supernatant was supplied to a second passage of the respective virus strains as described above (2^nd ^passage). For hantavirus and bornavirus the protocol was modified, as these usually induce no visible CPE.

#### Puumalavirus (PUUV)

BVK168 cells were infected with hantavirus Puumala strain Vranica. After incubation for 2 h at 37°C, unbound virus was removed by a triple washing and cells were incubated for the indicated time points at 37°C. The infection was monitored by the immunostaining of hantaviral N-protein or by the Western blot analysis of N-protein expression. For immunofluorescence, cells were fixed with acetone and stained with mouse monoclonal anti-Puumala N-protein (Progen, Heidelberg, Germany) and a secondary Cy3-conjugated anti-mouse antibody. For Western blot analysis, cells were lysed in SDS sample buffer, separated by SDS-PAGE and transferred to a nitrocellulose membrane. Hantaviral N-protein was detected using rabbit polyclonal anti-Puumala N-protein antibody [60]. Loading was controlled by the detection of tubulin on the same membrane with monoclonal anti-α-tubulin DM 1A (Sigma, Deisenhofen, Germany). Protein detection was performed using the super signal pico detection kit (Pierce, Bonn, Germany) according to the manufacturer's instructions. 9 and 12 days p.i. supernatants of BVK168 and VeroE6 cell lines were collected for the isolation of viral RNA with the RNeasy kit as described above. Isolated RNAs were analyzed by a light-cycler real-time RT-PCR targeting the S-segment (Weidmann M., unpublished).

#### Borna disease virus (BDV)

BVK168 cells grown in a 75 cm^2 ^flask were infected with BDV using a multiplicity of infection (MOI) of 0.1 by adsorbing 1 hour at 37°C in MEM with 2% FCS (BDV 1^st ^passage in BVK168). After an incubation time of 7 days in MEM with 10% FCS, the cells were harvested after trypsinisation in 5 ml MEM with 10% FCS. From this cell suspension 4 ml solution was pelleted by centrifugation at 400 g for 10 minutes, cells were resuspended in 1 ml MEM with 2% FCS, sonicated, clarified by centrifugation at 1000 g and assayed for infectivity. The remaining 1 ml cell suspension was inoculated in another 75 cm^2 ^flask with rabbit embryonic brain (REB) cells (BDV 2^nd ^passage in BVK168). After a further incubation time of 7 days the BDV-BVK168 cells were treated as described above in detail.

### Titration of viruses in BVK168 and back-titration in cell lines of origin

Virus titres of the 1^st ^and 2^nd ^passages on BVK168 cells were defined in parallel in the original cell line and in the BVK168 cell line. Briefly, after freeze-thawing the virus passages on BVK168 cell lines, serial dilutions of 10^0^-10^-8 ^were prepared in MEM containing 2% FCS. A 100 μl-aliquot of each virus dilution was inoculated in parallel in 48 hours-old-monolayers of BVK168 and the "original" cell lines (see table 1) in which the viruses were grown before (six well- plates, in duplicates). After incubation for 1 hour at 37°C, a 0.5% agarose in MEM buffered with NaHCO_3 _and 1xAAS was applied that was followed by a second 0.5% agarose overlay supplemented with neutral red at days 5 pi. For CPXV and VACV 1% methylcellulose was used instead of the agarose overlay and no neutral red was applied. Viral-induced plaques were counted at days 6 p.i. and titres expressed as plaque-forming units (Pfu)/100 μl.

BDV samples for infectivity assays were diluted from 10^-1 ^to 10^-8 ^in MEM plus 2% FCS. Portions of each dilution were mixed with equal volumes of freshly dispersed cultures of REB cells or BVK168 cells, inoculated in chamber slides (Lab-Tek Products, Naperville, Ill), incubated for 7 days at 37°C and fixed in acetone at -20°C. BDV titres were determined by an immunofluorescence assay as described previously [24].

## Results

### Isolated cells display epithelial morphology

Bank vole kidney cells showed outgrowth 10 days after preparation. 33 days post cultivation a first sub-passage was possible. Clonal outgrowth of singular cells was observed in the first sub-passage. From passage 5 on a splitting in ratio of 1:3 every 4 to 7 days was possible for sub-cultivation.

**Figure 1:**
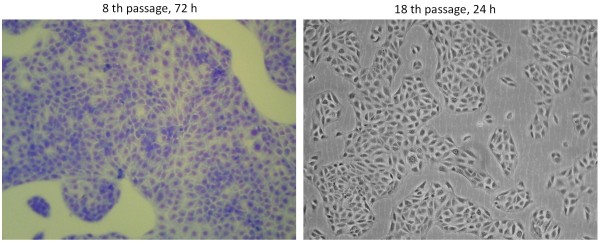
**Morphology of BVK168 cell line**. Subconfluent cells grown in MEM + 5% FCS were assessed for Romanowsky-Giemsa staining (8^th ^passage, 72 h) and phase contrast microscopy (18^th ^passage, 24 h). For figures of confluent cell lines see Figure 3.

**Figure 2:**
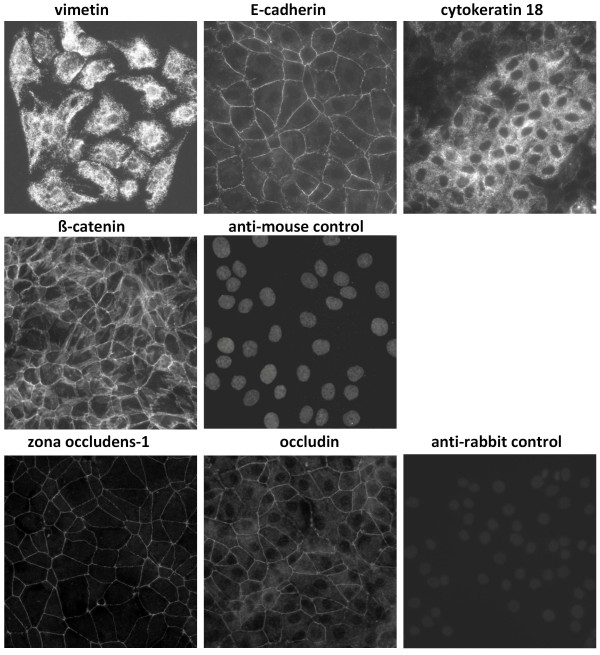
**Phenotypic characterization of BVK168 cells**. BVK168 cells were grown on coverslips, fixed and immunostained for the expression of marker proteins. Vimentin immunostaining displays parts of the cytoskeleton in the cytoplasm of BVK168. Immunostaining with the adhesions molecule and epithel cell marker E-cadherin is present at the cytoplasm membrane and Cytokeratin 18 as an epithelial intermediate filaments is visible in the cytoplasm of BVK168. ß-catenin staining is visible along the cytoplasm membrane into the cell as it is binding to the cell adhesion protein cadherine and connects this to the actin and cytoskeleton. Zona occludens-1 is at the cytoplasmic part and occludin a part of the epithelial tight junctions and therefore staining is visible at the BVK168 cytoplasm membrane. Negative controls stained with isotype controls for specifity of first antibodies with appropriate fluorescently-conjugated secondary antibodies are shown designated as anti-mouse control for vimetin, E-cadherin, cytokeratin 18, and ß-catein, and as anti-rabbit control for zona-occludens -1 and occludin.

BVK168 cells displayed the polygonal cobblestone-like morphology, which is typical for epithelial cells (Figure 1). Immunofluorescence microscopy was used to demonstrate the expression of different marker proteins on BVK168 cells. Cells were positive for the epithelial marker proteins vimentin, E-cadherin, and cytokeratin 18 (Figure 2). In contrast, cells did neither express the endothelial cell markers CD31 and von Willebrand factor nor the fibroblast-specific protein S100A (data not shown). In addition, the expression of components of intercellular junctions in confluent BVK168 cells was analyzed. Cells expressed the tight junction marker proteins ZO-1 (zonula occludens-1) and occludin and the adherens junction-proteins E-cadherin and β-catenin as was shown by immunofluorescence analysis (Figure 2). The localization of junctional proteins was exclusively at cell-cell contacts and exhibited a continuous staining along their boundaries.

The epithelial morphology of BVK168 and growth characteristics was maintained after continuous passaging and also after freeze-thawing of cell stocks.

### Genetic and antigenetic characterization

Serological investigation of the bank vole No. 168 showed OPV-neutralizing antibodies (SNT titre of 1:24), but no antibodies against hantaviruses or TBEV. In the PCR for OPV, Borrelia, Leptospira and mycoplasma none of the tested DNA sequences was amplified. The BVK168 cell line was free of analyzed nucleic acids specific for these agents, but - as would be expected for a cell type of vole origin - in the multiplex assay the internal mammal specific control PCR was positive. The derived partial mitochondrial cyt B sequences (996 nt) had highest homology (99%) to sequences from bank voles from other parts of Bavaria, South-East Germany (e.g. DQ090757; see [14]) and the Czech Republic (99% homology, Kasperske Hory DQ472293, and Ceske Budejovice DQ472294).

### BVK-168 is highly susceptible to different zoonotic viruses

**Figure 3:**
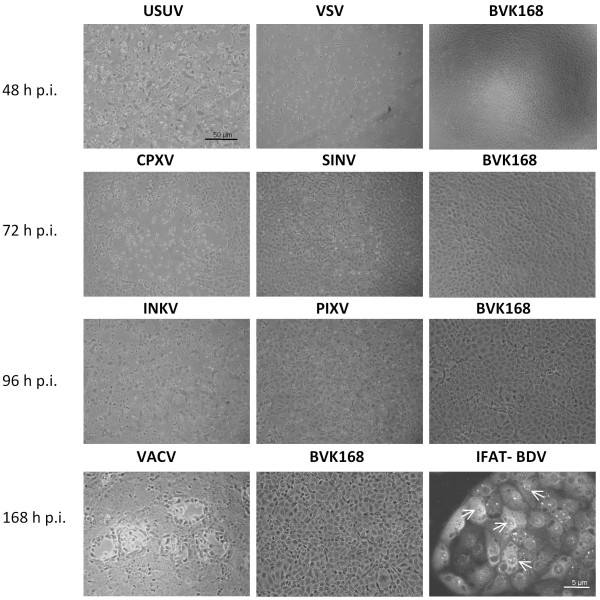
**Phase contrast of virus infected BVK168 cells and Immunofluorescence (IFAT) of Borna diseases virus (BDV) antigen in nuclei of BVK168 cells**. Phase contrast: Usutu virus (USUV) and vesicular stomatitits virus (VSV) are shown in comparison to uninfected BVK168 at 48 hours poist infectionem (h p.i.). Further Cowpox virus (CPXV), Sindbis virus (SINV) and uninfected BVK168 at 72 h p.i., Inkoo virus (INKV), Pixuna virus (PIXV) and uninfected BVK168 at 96 h p.i., Vaccinia virus (VACV) and uninfected BVK168 at 168 h p.i. are illustrated. Specific immunofluorescence of BDV is indicated with arrows.

The rhabdo-, orthopox-, alpha-, orthobunya- and flaviviruses chosen for the investigation of virus susceptibility all induced a cytopathic effect (CPE) in the BVK168 cell monolayer. Results of semi-quantitative evaluation of CPE are summarized in table 2. For VACV and CPXV (see Figure 3), the CPE was at the beginning characterized by the formation of plaques which later in the infection resulted in generalized CPE. For VSV, SINV, PIXV, INKV, and USUV (Figure 3) the CPE was more generalized, typical for these agents and comparable to that obtained in the original cell lines. Plaque titration showed that the viruses amplified to quite high titres for VSV, VACV, CPXV and INKV ranging from approximately 10^5 ^up to 10^6 ^Pfu/100 μl. USUV resulted in titres of 10^3 ^in the BVK168 cells and back-titration in Vero B4 cell lines up to 10^5 ^Pfu/100 μl. SINV had similar titres of 10^7 ^and PIXV of 10^8 ^in both BVK168 and Vero B4 cells. Detailed results of titres are given in table 3.

**Table 2:** Cytopathic effect of viruses in BVK168 cells.

BVK168 passage	**days p.i**.	VSV	VACV	CPXV	SINV	PIXV	INKV	USUV
1^st^	**1**	+	-	-	-	-	-	-
	**2**	+++	-	(+)	-	-	-	++
	**3**	++++, cf	-	++++, cf	(+)	(+)	-	++++, cf
	**4**	cf	(+)	cf	+	+	(+)	cf
	**5**	cf	(+)	cf	++	++	(+)	cf
	**6**	cf	(+)	cf	+++	+++	++++, cf	cf
	**7**	cf	++, cf	cf	++++, cf	++++, cf	cf	cf

2^nd^	**1**	+++	-	-	-	-	-	-
	**2**	++++, cf	-	(+)	(+)	-	-	++
	**3**	cf	++	++	+	(+)	(+)	++++, cf
	**5**	cf	+++	++++, cf	++	+	+	cf
	**5**	cf	+++	cf	+++	++	++	cf
	**6**	cf	++++, cf	cf	++++, cf	+++	+++	cf
	**7**	cf	cf	cf	cf	++++, cf	++++, cf	cf

Abbreviations: p.i., post infectionem; VSV, vesicular stomatitis virus strain India; VACV, vaccinia virus strain Munich 1; CPXV, cowpox virus strain 81/01; SINV, Sindbis virus strain C377; PIXV, Pixuna virus strain BeAr35645; INKV, Inkoo virus strain TN-98-5085; USUV; Usutu virus strain 0679/2006; -, no CPE; (+), 10-20% visible CPE; +, 20-40% visible CPE; ++, 40-60% visible CPE; +++, 60-80% visible CPE; ++++, >80% visible CPE; cf, culture frozen until further use

Viral cytopathic effect in BVK168 cells were evaluated semi-quantitative. Puumalavirus and Borna disease virus are not listed as these showed no visible CPE and evidence for infection of cells by these viruses had to be confirmed by real-time RT-PCR and/or immunofluorescence assay.

**Table 3:** Results of virus titrations on BVK168 cells.

BVK168 passage	Titer (Pfu/100 μl)	VSV	VACV	CPXV	SINV	PIXV	INKV	USUV
1^st^	BVK168	4.5 × 10^6^	7 × 10^6^	4.9 × 10^5^	2 × 10^7^	1 × 10^8^	7 × 10^5^	1.6 × 10^3^
	Original line*	4.5 × 10^5^	1 × 10^6^	7 × 10^5^	2 × 10^7^	1 × 10^8^	1 × 10^5^	1 × 10^5^

2^nd^	BVK168	2 × 10^6^	6.5 × 10^6^	2.64 × 10^6^	n.d.	n.d.	7.2 × 10^5^	8.2 × 10^3^
	Original line*	4.8 × 10^5^	3 × 10^6^	1.4 × 10^6^	n.d.	n.d.	2.2 × 10^5^	1 × 10^5^

* original cell lines are shown in table 1

Abbreviations: p.i., post infectionem; Pfu, plaque forming units; VSV, vesicular stomatitis virus strain India; VACV, vaccinia virus strain Munich 1; CPXV, cowpox virus strain 81/01; SINV, Sindbis virus strain C377; PIXV, Pixuna virus strain BeAr35645; INKV, Inkoo virus strain TN-98-5085; USUV; Usutu virus strain 0679/2006; cf, culture frozen until further use; n.d., not determined

Titers gained on BVK168 cells in comparison to back-titration on original cell line. Puumalavirus and Borna disease virus are not listed as these showed no visible CPE and evidence for infection of cells by these viruses had to be confirmed by real-time RT-PCR and/or immunofluorescence assay.

BVK168 cells inoculated with BDV did not induce a visible CPE. However, immunofluorescence revealed that 7 days p.i. approximately 25% of the cells were infected, and after the first cell passage about 90%. The infected cells showed the typical granular fluorescence in the nucleus (Figure 3). The infectivity assay revealed identical titres of 6 × 10^5 ^ID_50_/ml for the first passage and 2 × 10^6 ^ID_50_/ml for the second passage in REB cells and BVK168 cells.

**Figure 4:**
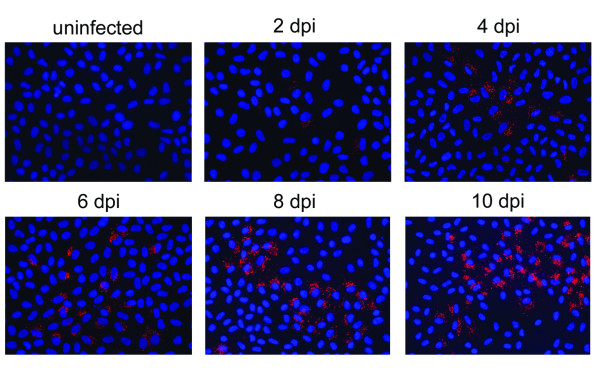
**Immunofluorescence of Puumala virus infected BVK168 cells**. BVK168 cells were infected with Puumala virus (PUUV) or left uninfected. At the indicated time points, cells were fixed and stained for N-protein. Nuclei were stained with Hoechst 33342.

**Figure 5:**
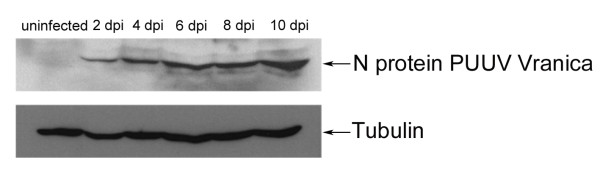
**Expression of Puumala virus N-protein in infected BVK168 cells**. Cell lysates were analyzed for expression of N-protein and tubulin.

**Figure 6:**
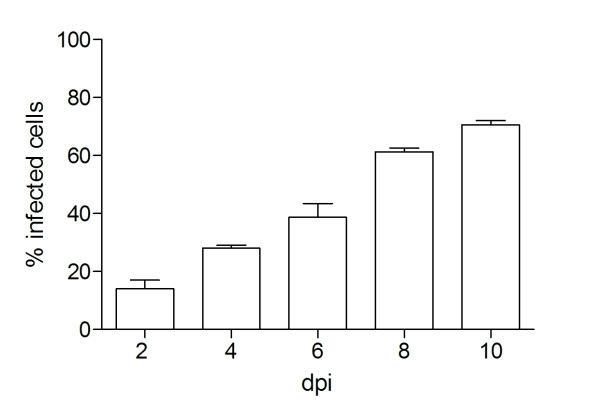
**Quantification of Puumala virus N-protein in infected BVK168 cells**. Infected cells were quantified by counting N-protein-expressing cells. Shown data are representative for three independent experiments. Error bars represent the standard deviation.

**Figure 7:**
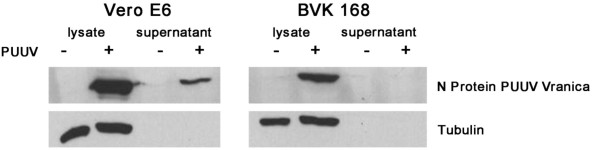
**Non-productive infection of BVK168 cells with PUUV**. Supernatants of infected Vero E6 and BVK168 cells were analyzed for the presence of the hantaviral N protein.

**Figure 8:**
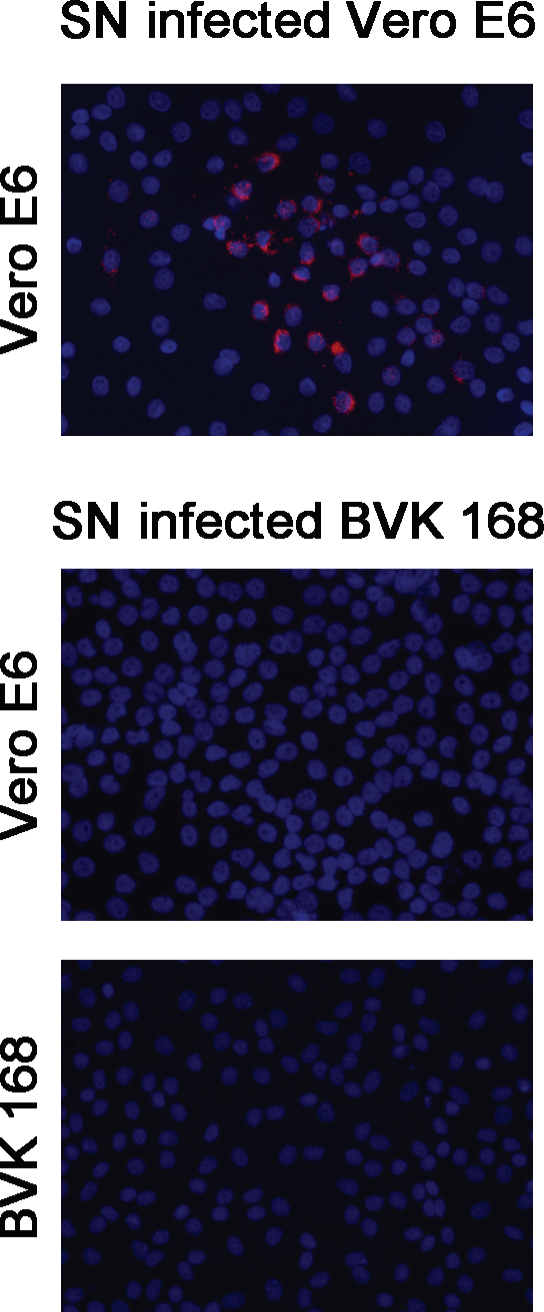
**Reinfection of Vero E6 and BVK168 cells with supernatants of infected Vero E6 and BVK168 cells**. Vero E6 and BVK168 cells were incubated with supernatant of PUUV-infected BVK168 cells. As a control Vero E6 were incubated with supernatant of infected Vero E6 cells. Infected cells were detected by staining of hantaviral N protein (SN, supernatant).

To assess the susceptibility of BVK168 cells to Puumala virus, we analyzed cells incubated with Puumala Vranica for the expression of hantaviral N protein by immunofluorescence (Figure 4) and Western Blot (Figure 5). Quantification of infected cells revealed that about 60% of the cells are infected eight days post infection (Figure 6). We next examined whether BVK168 cells produce infectious hantavirus particles. In the Western Blot analysis of cell culture supernatant of infected BVK168 cells we could not detect hantaviral N protein (Figure 7) and reinfection of Vero E6 or BVK168 cells with supernatant (SN) of infected BKV168 cells was not possible (Figure 8). PUUV RNA was present in the cellular lysate as revealed by real-time RT-PCR (12 days p.i., cycle threshold, CT 24). However, in the cell culture medium we could not detect viral RNA (9 and 12 days p.i. no CT). In comparison, supernatants of VeroE6 cells infected with PUUV showed a negative shift of 12 CT values, estimated to be equivalent to an increase of approximately 4 log steps in viral titre. In summary, these results demonstrate that the infection of BVK168 cell with this hantavirus is transcriptionally active but non-productive.

## Discussion

Approximately 75% of the emerging pathogens originate from wildlife animals. Retrospective studies performed worldwide on data from 1940 to 2004 have shown a continuing upward trend [1,2]. Due to the biological relevance of their host species, rodent-borne pathogens beneath bat-associated viruses seem to play the most important role in the emergence of pathogens [5,32,33]. This hypothesis seems to be supported by the ongoing reports of newly described rodent-associated agents such as Ljunganvirus or new herpesviruses. Sufficient wild rodent cell culture models are needed in order to enable reliable investigation of these pathogens. Among the rodent species and associated pathogens described in Europe, *Myodes glareolus *belongs to some of the most relevant ones. In this paper we therefore describe the establishment of a cell line from kidneys of *Myodes glareolus.*

The appearance of the BVK168 cell line, which was isolated from bank vole kidney by single cell cloning, showed a typical epithelial morphology. The epithelial phenotype was further confirmed by the immunostaining of differentially expressed proteins. Intermediate filament proteins and adhesion molecules serve as markers for specific cell types. Cytokeratins are almost exclusively expressed in epithelia [34,35]. Loss of cytokeratin and E-cadherin expression and up-regulation of vimentin expression in epithelia correlates with decreased differentiation and epithelial to mesenchymal transition (EMT). However, co-expression of cytokeratin and vimentin was also observed in normal epithelium without signs of dedifferentiation or EMT particularly in epithelia derived from the urogenital tract and in cultured epithelial cell lines [36]. The transformation of epithelial cells to a fibroblastic phenotype is accompanied by the downregulation of tight and adherens junction-proteins [37]. As demonstrated by immunofluorescence, BVK168 cells express the epithelial marker proteins cytokeratin 18 and vimentin. The tight junction-proteins ZO-1, occludin and the adherens junction-proteins E-cadherin and β-catenin are expressed and localized at the cell-cell contacts. In contrast, BVK168 cells did not express proteins specific for endothelial cells or fibroblasts. Therefore, the characterization of the BVK168 cell line reveals a typical epithelial phenotype.

The established BVK168 cell line has so far successfully been propagated to the 49^th ^passage without the need of an immortalization procedure. Spontaneous rodent cell immortalization has been previously reported from many mouse cell lines of different tissue origin and the underlying mechanisms have been investigated for some of these lines [38-42].

The established cell line was highly susceptible to nine very different lab-adapted virus strains of six virus families. For the rhabdovirus VSV and the poxviruses VACV and CPXV it is well known that these viruses have a broad host range *in vitro*. VSV and CPXV can be grown on a variety of cell lines, e.g. different mammalian or mosquito cells, embryonated chicken eggs, and suckling mice or weanling mice [43-45]. For VACV it was further described that it even propagates in amphibian cells such as frog neurons [46,47]. Our results for BVK168 cells are therefore in line with these findings.

Following the emergence of USUV in Austria in 2001, a detailed analysis of viral multiplication in 13 permanent cell lines, 3 primary cell cultures, and chicken embryos was published [48]. While chicken embryo fibroblast cells and chicken embryos were resistant to infection, the flavivirus induced a visible cytopathic effect in Vero, PK-15, and goose embryo fibroblast cells. However, in other cell types as hamster (BHK-21, BF), rat (C6) cell lines virus antigen only could be shown by immunohistochemical tests [48]. Given this context, it is remarkable to observe a generalized CPE in BVK168 cells for USUV.

SINV as the alphavirus prototype has been intensively studied for its growth characteristics in a broad range of vertebrate and also invertebrate cells with barely a cell line which did not support its propagation [18,49]. Susceptibility of cell lines for Inkoo virus was shown for baby hamster kidney, mosquito and renal African green monkey cell lines [21]. Viruses of the California encephalitis serogroup such as Inkoo virus are pathogenic for suckling lab mice and have serologically been detected in bank voles in nature [20,21]. PIXV was shown to infect mouse embryo cells [50], spiny rat (*Proechimys semispinosus*) with low viremia [19] and to induce pathogenic changes in brain and spleen of white mice [21,51]. Propagation of PIXV and Inkoo virus in BVK168 cells is therefore in line with results of growth in rodents or rodent cell lines described previously.

Surprisingly, propagation of PUUV strain Vranica in BVK168 failed although this virus strain originally derived from bank voles. This was tested for low and high passages of BVK168 (data not shown in detail) and failed in all performed experiments. The method of choice for isolation of PUUV from rodents currently is its propagation in bank voles in colony [5,52]. For the strain Kazan it was shown that a Vero E6-adapted strain replicated in these cells to a high efficiency, but did not reproducibly infect bank voles any more. These findings were a consequence of the accumulation of point mutations in S and L gene [53,54]. This might also be true for the PUUV strain Vranica used in this study. PUUV Vranica is also well adapted to Vero E6 tissue cultures and may have undergone mutations during adaption. Trials to isolate PUUV from PUUV-positive bank voles from South Bavaria [55] in different passages (8^th^, 16^th^, 35^th ^passage) of BVK168 in parallel with Vero E6 cell lines by several sub-passages failed (data not shown). Generally, hantaviruses grow slowly and require up to 10 days to reach 100% infection depending on the adaptation level of the virus strain, infectious doses, and the cell type used. Isolation of hantaviruses proved to be often difficult, requiring several blind passages of the inoculated Vero E6 cell cultures during which the virus adapts to cell culture. The molecular mechanism and genetic basis of the slow growth and adaptation of the virus in cell culture are poorly understood. We revealed no PUUV propagation in the established BVK168 cell line. In comparison, Temonen & coworkers [14] could show that out of several human cell lines and established bank vole primary cultures exclusively primary bank vole kidney cells could propagate the PUUV virus strain Sotkamo as efficiently as Vero E6 cells with approximately 80% positive cells at day 7 p.i.. However, primarily isolated kidney cells are a pool of different cell types from this tissue. Therefore these may have quite different properties than our BVK168 cell line that has been established by clonal outgrowth of kidney cells. Although meanwhile there exist data on the urine excretion of PUUV in experimentally infected bank voles [56], to the authors' knowledge the mechanism of propagation of the virus in the voles kidney and the relevant cells have not been investigated in detail. Infections in humans are well known to take place in macrophages and vascular endothelial cells of kidney and lung [57,58]. As the established BVK168 cells were gained by clonal outgrowth, we cannot exclude that these represent a cell type of vole kidneys that cannot be infected with PUUV. Further experiments e.g. also including antibodies against non-structural proteins or cell compartments have to prove where the block during replication of PUUV in BVK168 is elapsing.

Many epidemiological facts point towards small rodents as BDV reservoir hosts [11]. However, BDV was not yet isolated from naturally infected rodents. Only in one Eulipotyphla (former insectivores) species, the bicolored white-toothed shrew (*Crocidura leucodon*), BDV could be detected by TaqMan real-time RT-PCR and by immunohistology without evidence of inflammation or degenerative processes in the brain [12]. So far multiple studies have been performed to show the presence of infectious BDV in tissues or blood of human or animal patients. However there are limited methods for the detection of BDV and also only few cell cultures for propagation of BDV [59,60]. Therefore the new established BVK168 cell lines also could be implemented in future investigations of BDV in neurological human disorders.

## Conclusions

In conclusion, our BVK168 cell line will be an excellent model to study infections of rodent-borne pathogens in original host cells *in vitro*. Further studies using new or difficult-to-culture rodent-associated viruses will have to proof suitability of the BVK168 cell line in that regard.. The BVK168 cell line will be a comprehensive tool for the investigation of pathogenesis of rodent-borne viruses for which relevant *in vitro *cell culture models are still lacking. This offers also a comparison of the underlying growth characteristics, pathogenesis mechanisms, and pathways of cell death (e.g. apoptosis) in rodent and other vertebrate host cells.

## Competing interests

The authors declare that they have no competing interests.

## Authors' contributions

SSE established the cell line, performed the experiments with pox-, flavi-, bunya-, rhabdo- and togaviruses, and wrote the manuscript draft. EK performed experiments with epithelial cell markers and PUUV, and revised the manuscript. SH performed the experiments with Borna disease virus and revised the manuscript. MP was involved in all experiments and contributed to the manuscript. All authors read and approved the manuscript.
